# *Francisella tularensis* subsp. *tularensis* Induces a Unique Pulmonary Inflammatory Response: Role of Bacterial Gene Expression in Temporal Regulation of Host Defense Responses

**DOI:** 10.1371/journal.pone.0062412

**Published:** 2013-05-14

**Authors:** Kathie-Anne Walters, Rachael Olsufka, Rolf E. Kuestner, Ji Hoon Cho, Hong Li, Gregory A. Zornetzer, Kai Wang, Shawn J. Skerrett, Adrian Ozinsky

**Affiliations:** 1 Institute for Systems Biology, Seattle, Washington, United States of America; 2 Division of Pulmonary and Critical Care Medicine, University of Washington, Seattle, Washington, United States of America; Karolinska Institutet, Sweden

## Abstract

Pulmonary exposure to *Francisella tularensis* is associated with severe lung pathology and a high mortality rate. The lack of induction of classical inflammatory mediators, including IL1-β and TNF-α, during early infection has led to the suggestion that *F. tularensis* evades detection by host innate immune surveillance and/or actively suppresses inflammation. To gain more insight into the host response to *Francisella* infection during the acute stage, transcriptomic analysis was performed on lung tissue from mice exposed to virulent (*Francisella tularensis* ssp *tularensis* SchuS4). Despite an extensive transcriptional response in the lungs of animals as early as 4 hrs post-exposure, *Francisella tularensis* was associated with an almost complete lack of induction of immune-related genes during the initial 24 hrs post-exposure. This broad subversion of innate immune responses was particularly evident when compared to the pulmonary inflammatory response induced by other lethal (*Yersinia pestis*) and non-lethal (*Legionella pneumophila, Pseudomonas aeruginosa*) pulmonary infections. However, the unique induction of a subset of inflammation-related genes suggests a role for dysregulation of lymphocyte function and anti-inflammatory pathways in the extreme virulence of *Francisella*. Subsequent activation of a classical inflammatory response 48 hrs post-exposure was associated with altered abundance of *Francisella*-specific transcripts, including those associated with bacterial surface components. In summary, virulent *Francisella* induces a unique pulmonary inflammatory response characterized by temporal regulation of innate immune pathways correlating with altered bacterial gene expression patterns. This study represents the first simultaneous measurement of both host and *Francisella* transcriptome changes that occur during *in vivo* infection and identifies potential bacterial virulence factors responsible for regulation of host inflammatory pathways.

## Introduction

*Francisella tularensis* is a gram-negative, facultative intracellular bacterium that can cause life-threatening zoonotic infections in humans. There are four primary subspecies of *F. tularensis*: *F.tularensis* subsp. *mediasiatica* and *novicida*, both of which are attenuated in humans, *F*. *tularensis* subsp. *holarctica*, which causes a mild disease in humans and *F*.*tularensis* subsp. *tularensis*, which causes a severe disease in humans and other mammals [1], [2]. Transmission can occur through multiple routes including ingestion of contaminated food or water, arthropod vector, direct contact through mucosa or broken skin, or through inhalation of bacteria [3], [4]. Aerosol exposure to *F. tularensis* is associated with the most severe disease, with mortality rates as high as 30% with the *F. tularensis* subsp. *tularensis* (Type A) strain, if left untreated [5]. Type A *F. tularensis* is also particularly infectious, with as few as 10 organisms capable of causing human disease [4]. It is for this reason that Type A *F. tularensis* has been classified as a Category A select agent by the Centers for Disease Control and Prevention and is considered a potential bioweapon, leading to increased interest in understanding the pathogenesis of this infection [6].

The host-bacteria interaction is a complex process, involving both the detection of the bacteria by the host as well as manipulation of host biological pathways by the bacterium in an attempt to facilitate bacterial replication/dissemination. The extreme virulence of *F. tularenensis* subsp. *tularensis* is not well understood but multiple studies, in humans and mice, have shown that *Francisella* infection is associated with the absence of a classical host innate immune response (reviewed in [7]. In seven individuals with ulceroglandular tularemia, a decreased expression of genes (relative to samples from normal controls) involved in innate and adaptive immune responses is seen in peripheral blood during the acute phase of infection [8]. In mice, *F. tularensis* fails to stimulate production of classical pro-inflammatory mediators, including TNF-α, and IL-12p40, in the airways and lungs of aerosol-exposed animals and fails to activate dendritic cells (DC) [9]. Induction of Type II interferon is also impaired during *F. tularensis* infection of mononuclear phagocytic cells [10]. Similarly, infected human DCs/macrophages also demonstrated a lack of induction of multiple cytokines [11]–[14]. Interestingly, both infected and adjacent uninfected DCs fail to respond to Toll-like receptor agonists, suggesting *F*. *tularensis* is actively suppressing activation of targeted inflammatory responses through intracellular molecules [9], [12]. Collectively, these studies demonstrate that *F.tularensis* does not induce a classical pro-inflammatory immune response. However, most of these investigations have focused on a limited set of inflammatory mediators and so the extent of this suppression with respect to the broader immune response is yet to be fully characterized. Moreover, many of the studies have utilized *F. tularensis* subspecies that do not exhibit the extreme virulence of the *F. tularensis* ssp. *tularensis* strain.

The current study focused on characterizing the extent of the suppression of the host pulmonary inflammatory response caused by virulent Group A *F. tularensis* SchuS4 (FT SchuS4) through detailed transcriptomic study of both host and pathogen. The host response to FT SchuS4 was also compared to that of the highly lethal *Yersinia pestis*, attenuated strains of *F. tularensis* and *Y. pestis*, as well as *Pseudomonas aeruginosa* and *Legionella pneumophila*, which are other respiratory pathogens that induce a severe but non-lethal infection. While acute *F. tularensis* infection at four hours was associated with a marked suppression of multiple aspects of the innate immune response relative to other pathogens, a subset of immune-related transcripts were uniquely induced by virulent *F. tularensis*, including those that function to modulate inflammation. A classical inflammatory response was activated in *Francisella*-infected mice after 24 hrs which correlated with a dramatic change in bacterial gene expression patterns. This simultaneous measurement of host and bacterial transcriptome changes that occur during *in vivo* infection identified potential virulence factors which target host inflammatory pathways.

## Materials and Methods

### Bacteria

*Francisella tularensis* subspecies *tularensis* SchuS4 (CDC, Fort Collins, CO) was grown to stationary phase with agitation at 37°C in Mueller Hinton broth supplemented with 2% Isovitalex (BBL), pelleted, suspended in PBS with 20% glycerol, aliquoted, and stored at −80°C. The post-freeze titer of this stock was 3×10^9^ CFU/ml when cultured on cysteine heart agar supplemented with 2% hemoglobin. *Francisella tularensis* subspecies *holarctica* live vaccine strain (LVS) was obtained from Karen Elkins (FDA, Rockville, MD). Bacteria were grown to stationary phase in Mueller Hinton broth supplemented with 2% Isovitalex, 1% glucose, and 0.25% ferric pyrophosphate, washed, suspended in PBS with 20% glycerol, aliquoted, and stored at −80°C. The post-freeze titer of this stock was 4×10^9^ CFU/ml when cultured on modified Mueller Hinton agar supplemented with 2% hemoglobin and 1% Isovitalex. *Yersinia pestis* CO92 and avirulent *Y. pestis* CO92 that had been cured of the major virulence plasmid pCD1 that encodes the virulence antigen (Lcr or V) were obtained from Joseph Hinnebusch (Rocky Mountain Laboratories, Hamilton, MT). Virulent *Y. pestis* was grown to stationary phase in brain heart infusion broth, pelleted, suspended in PBS with 20% glycerol, aliquoted, and stored at −89°C. The post-freeze titer of this stock was 10^10^ CFU/ml when cultured on sheep's blood agar. Avirulent *Y. pestis* was grown to stationary phase in LB broth, pelleted, suspended in PBS with 20% glycerol, aliquoted, and stored at −80°C. The post-freeze titer of this stock was 2×10^9^ CFU/ml when cultured on LB agar. *Pseudomonas aeruginosa* strain PAK was obtained from Stephen Lory (Harvard University, Cambridge, MA). Bacteria were grown to stationary phase in LB broth, diluted with 20% glycerol, aliquoted, and stored at −80°C. *Legionella pneumophila*, Philadelphia 1 (ATCC 33152) was grown to stationary phase in buffered yeast extract broth, diluted with 20% glycerol, aliquoted, and stored at −80°C.

### Mice

Male and female BALB/c mice 8–10 weeks of age and free of specific pathogens were purchased from Jackson Laboratories (Bar Harbor, ME). Mice were housed in laminar flow cages and permitted ad lib access to sterile food and water. Animal studies were conducted in compliance with the National Research Council Guide for the Care and Use of Laboratory Animals. The mouse experiments were specifically approved by the University of Washington Institutional Animal Care and Use Committee, under protocol numbers 2671-06 and 2982-03.

### Mouse exposures and tissue harvests

Mice were exposed to aerosolized bacteria in a nose-only inhalation chamber (In-Tox products, Moriarty, NM) using a computer interface to control pressures and flows (Biaera Technologies, Frederick MD). Aerosols were generated by mini-Heart nebulizers with a flow rate of 8 L/min at 40 psi. Dilution air was regulated at 11.5 L/min to maintain total chamber flow at 19.5 L/min during a 10-minute exposure. *F. tularensis* SchuS4 and *Y. pestis* CO92 were aerosolized at concentrations demonstrated in preliminary studies to result in similar bacterial depositions in the lungs that were uniformly lethal within 5 days. *F. tularensis* LVS was aerosolized at a concentration that was shown in preliminary studies to result in uncontrolled bacterial replication in the lungs and dissemination to other organs. The depositon of *F. tularensis* LVS in the lungs after aerosol exposure in these experiments was approximately 10-fold higher than the reported lethal dose for BALB/c mice infected by the aerosol route [15], [16]. For infection with *P. aeruginosa* or *L. pneumophila*, bacteria were freshly grown in broth to stationary phase, washed, and suspended in PBS for nebulization. These infections are non-lethal by the aerosol route and bacteria were nebulized at concentrations that were known to produce a vigorous inflammatory response and transient though significant clinical illness [17], [18]. Bacterial deposition in the lungs was determined in each experiment from quantitative culture of lung tissue harvested from two sentinel mice immediately after exposure. Measured depositions for all pathogens were as follows: *F. tularensis* subsp. *tularensis* (SchuS4) (487, 447 CFU/lung), *F. tularensis* subsp. *holarctica* (LVS) (2.7×10^4^, 3.0×10^4^ CFU/lung), virulent *Y. pestis* (561, 266 CFU/lung), avirulent *Y. pestis* (2.0×10^4^, 1.6×10^4^ CFU/lung), *P. aeruginosa* (8.9×10^5^, 5.9×10^5^), *L. pneumophila* (4.7×10^5^, 4.5×10^5^). Control mice were exposed to aerosolized PBS. For each bacterial and PBS challenge, at least two separate experiments were performed. Mice were monitored daily for visible signs of illness (alterations in activity, eyes, coat, respiratory pattern, and posture) and ventral surface temperature was monitored with an infrared thermometer (Raytek). To characterize the early course of each infection, 3–4 mice were euthanized after 4, 24 and 48 hrs with intraperitoneal pentobarbital and exsanguinated by external cardiac puncture. Left lungs, spleens, and left lobes of the liver were homogenized in PBS for quantitative cultures on appropriate solid media and measurement of cytokines. The right lung was lavaged with 0.9% sodium chloride containing 0.6 mM EDTA, then inflated and fixed in 4% paraformaldehyde, as described [18]. The pooled lavage fluid from each mouse was pelleted for measurement of cell counts and differentials, as previously described [18]. Fixed lung tissue was embedded in paraffin, sectioned, and stained with hematoxylin and eosin. Separate experiments were performed for RNA extraction. Mice were infected and euthanized in the same manner as in the characterization experiments. After 4, 24, and 48 h the pulmonary arteries were perfused with 5 ml cold PBS and the lungs were homogenized in Qiazol extraction solution (Qiagen). Total RNA was purified with Qiagen miRNA spin columns. The RNA concentration was measured using a Nanodrop spectrophotometer and the quality was checked with a Bioanalyzer. To determine the effect of *Francisella* infection on responsiveness to a TLR4 ligand, mice were exposed to aerosolized *E. coli* 0111:B4 LPS (List) at a concentration of 100 µg/ml 18 h after inhalation of FT SchuS4, FT LVS, or PBS. After 2.5 h lung RNA was harvested as described above.

### RNA isolation and expression microarray analysis

Total RNA was isolated from lung by homogenization (10% w/v) in Trizol (Invitrogen, Carlsbad, CA) followed by chloroform extraction and isopropanol precipitation. RNA quality was assessed using a BioAnalyzer (Agilent Technologies, CA). Gene expression profiling experiments were performed using Agilent Mouse Whole Genome 44K microarrays. Fluorescent probes were prepared using Agilent QuickAmp Labeling Kit according to the manufacturer's instructions. Each RNA sample (4 biological replicates per condition) was labeled and hybridized to individual arrays. Spot quantitation was performed using Agilent's Feature Extractor software and all data were then entered into a custom-designed database, SLIMarray (http://slimarray.systemsbiology.net), and then uploaded into Genedata Analyst 7.0 (Genedata, Basel, Switzerland). Data normalization was performed in Genedata Analyst using central tendency followed by relative normalization using pooled RNA from mock infected mouse lung as reference. Transcripts showing differential expression (2-fold, p value<0.01) between infected and control animals were identified by standard t test. The Benjamini-Hochberg procedure was used to correct for false positive rate in multiple comparisons. Metacore version 6.3, Entrez Gene (www.ncbi.nlm.nih.gov/sites) and NextBio (www.nextbio.com) were used for mammalian gene ontology and pathway analysis. DAVID Bioinformatic Resources 6.7 was used for bacterial gene ontology (david.abcc.ncifcrf.gov). The complete microarray dataset has been deposited in NCBI's Gene Expression Omnibus [19] and is accessible through GEO Series accession number GSE44320.

### Quantitative RT-PCR

Quantitative real-time PCR was used to estimate bacterial loads in lung tissue. Reverse transcription of total lung RNA was performed using either gene-specific primers or random primer and the Superscript III First Strand cDNA synthesis kit (Invitrogen, Carlsbad, CA). Taqman primers and probe were designed to the *L. pneumophila* and *P. aeruginosa* 16S rRNA gene using the PrimerQuest design tool (Integrated DNA Technologies) generating the following primers and probes: *L. pneumophila*: forward (5′-CAAGTCGAACGGCAGCATTGTCTA -3′), reverse (5′-GCGTTACTCACCCGTTCG-3′), probe (5′-GCTTGCTAGACAGATGGCGAGTGG-3′), *P. aeruginosa*: forward (5′-CTAACACATGCAAGTCGAGCGGAT-3′), reverse (5′-GCATTACTCACCCGTCCGCC-3′), probe (5′-AAAGGGAGCTTGCTCCTGGATTCA-3′). Primers and probes used for detection of *F. tularensis* and *Y. pestis* strains were taken from [20].

Real-time PCR was performed using an ABI 7900HT Fast Real Time PCR system. Each target was run in duplicate with Taqman 2X PCR Universal Master Mix and a 5-µL total reaction volume. Endogenous control primer and probe sets for relative quantification included either GAPDH mRNA or 18S ribosomal RNA and were obtained from Applied Biosystems, Foster City, CA. Quantification of CT for each gene was graphed relative to that of the calibrator: mouse GAPDH (ABI #4352932-0708015) and mammalian 18S rRNA (ABI # HS99999901_s1).

### Library preparation for Illumina RNA-seq

Ten micrograms of total RNA isolated from lung tissue (equal amounts pooled from 3 individual animals) was sequentially treated with MICROBEnrich and MICROBExpress kits (Ambion) to enrich for bacterial mRNA through removal of mammalian mRNA/rRNA and bacterial rRNA, respectively. Two sequential rounds of MICROBEnrich were performed followed by one round of MICROBExpress according to the manufacturer's instructions. The resultant bacterial mRNA was fragmented using a RNA fragmentation kit (Ambion), which yielded fragments of 60–200 nucleotides in length. Libraries for Illumina sequencing were prepared as recommended by Illumina.

### RNA-seq data analysis

Raw sequence files were pre-processed by NGSQCToolkit v2.2.3 for quality control, which removes adapter and filters low quality sequences for which the quality score of more than 70% bases is lower than 20. To determine the identity of the sequence reads, the processed sequence files were mapped to the *Mus musculus* genome using Tophat v1.4.0. Unmapped reads were then aligned to the genome of *Francisella tularensis* SchuS4 strain. Mapped reads were input into Cufflinks v1.3.0 (http://cufflinks.cbcb.umd.edy/) to estimate the relative abundances of transcripts. Sequencing data are available in the ArrayExpress database (www.ebi.ac.uk/arrayexpress) under accession number E-MTAB-1571.

## Results

### Virulent *Francisella tularensis* induces an extensive early host transcriptional response in the lungs of animals

Animals were exposed via aerosol to virulent *Francisella tularensis* ssp. *tularensis* (FT SchuS4) to investigate the inflammatory response to pulmonary *F. tularensis* infection, which is associated with the most severe form of tularemia. The dose used in these experiments (see Materials and Methods) was uniformly fatal by day five. Lung tissue was harvested at four, 24 and 48 hrs post-exposure and global gene expression profiling was performed. For each experiment, mRNA from an individual infected animal was compared to mRNA from a pool of PBS-exposed control animals (n = 9).

**Figure 1:**
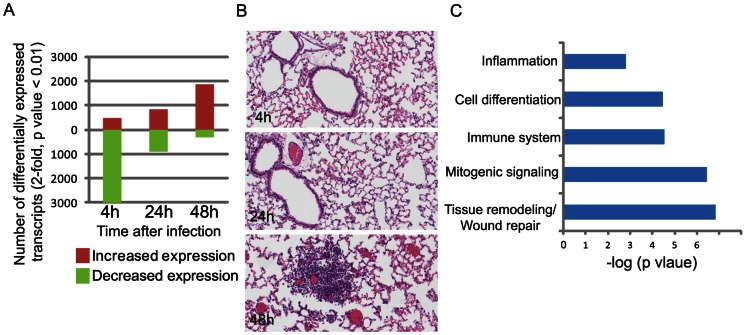
Global host transcriptional response in the lungs of animals following aerosol exposure to virulent *F.* tularensis. **A**. Number of transcripts that are differentially expressed (at least 2-fold difference in median expression level, p value 0.01) in the lungs of animals exposed to FT SchuS4 at 4, 24 and 48 hrs post-exposure. Transcripts showing increased or decreased expression relative to control animals are shown in red and green bars, respectively. B. Hematoxylin and eosin stained lung tissue from animals exposed to FT SchuS4. Magnification 20x. **C.** Gene ontology analysis of approximately 400 transcripts induced by FT SchuS4 at 4 hrs demonstrating enrichment of specific functional groups.

Surprisingly, an extensive host transcriptional response was observed in animals exposed to FT SchuS4 as early as four hours post-exposure, with over 3000 transcripts showing a significant change in expression level (at least 2-fold difference in median expression level, p-value<0.01) relative to control animals at this time-point (Figure 1A). In previous studies, a less extensive transcriptional response to inhaled *Francisella* was observed when lower exposure doses and different microarray platforms were used [21], [22]. At four hours post-exposure, animals exhibited no observable signs of illness and the bacterial load did not measurably increase (data not shown). Furthermore, little to no histological changes were observed in the lungs at either 4 or 24 hrs (Figure 1B), although by 48 hrs there were multiple foci of intense alveolar and perivascular necrotizing neutrophils inflammation (Figure 1B).

The change in expression levels of over 3400 transcripts four hours post-FT SchuS4 exposure demonstrate that host signaling pathways recognize and respond to the presence of the bacteria. Gene ontology enrichment analysis on the approximately 400 transcripts showing increased expression at this time-point revealed the involvement of the immune response and inflammation, in addition to cell differentiation and mitogenic signaling (Figure 1C). Also induced were genes associated with tissue remodeling, including cadherin-mediated cell adhesion (PTPRF, PTPN11, FERT2, Slk, Fmn1), which links cell surface adhesion and recognition molecules to the actin cytoskeleton and intracellular signaling pathways. Consistent with this was enrichment of genes related to cytoskeleton remodeling/intracellular trafficking (including PIK3R1, PIK3CB, Slk, Tln2, Rock1, Afap1, Cgn, Myolb, Myo9a, Myo6, Plec1, Spire1,Tln2, Twf1, Vapb, Wasf2, Ckap4/5, Plcb1) and MAP kinase signaling signaling (Map3k4, Map4k3, Map4k4, Mapk6, Dusp7/4/18). Collectively, these results suggest that the host detects and responds to FT SchuS4 early after exposure, including those processes associated with infection.

### *F. tularensis* induces a limited pulmonary inflammatory response during acute infection relative to other respiratory pathogens despite active bacterial replication

Gene ontology analysis of sequences showing increased expression during acute FT SchuS4 infection suggested that host immune response pathways are responding to the presence of the bacteria, at least to some extent. Previously, studies examining the lack of activation/active suppression of inflammation by *Francisella* have been primarily focused on a limited set of well-known mediators, such as TNFα, IL6, IL1β, etc, as a measurement of immune response to infection. Furthermore, while comparison of virulent and attenuated *Francisella* strains has been done [23], comparison of FT SchuS4-mediated immune response to that induced during a severe but non-lethal respiratory infection has not been explored. To better understand the nature of the *Francisella*-associated inflammatory response relative to that induced by other lethal as well as non-lethal respiratory infections, animals were exposed via aerosol to a lethal dose of an attenuated strain of *Francisella* (FT LVS), a lethal dose of *Y. pestis* (YP), a non-lethal dose of a plasmid-deficient avirulent strain of *Y. pestis* (aYP), a non-lethal dose of an intracellular pathogen *L. pneumophila* (LP), or to a non-lethal dose of the extracellular pathogen *P. aeruginosa* (PA). Mice were infected with FT LVS at a concentration that was approximately 10-fold higher than the reported median lethal dose [15], [16] and which resulted in uncontrolled bacterial replication in our preliminary studies (not shown). Virulent YP was dosed at a concentration similar to that used for FT SchuS4, and demonstrated in preliminary studies to be uniformly lethal within 5 days (data not shown). The avirulent strain of YP was dosed at an arbitrary concentration approximately 50-fold higher than the virulent strain, and resulted in no visible illness. LP and PA were dosed at concentrations known to produce vigorous inflammatory responses and transient clinical illness; both of these infections are non-lethal by the aerosol route in mice [17], [18]. At 4, 24 and 48 hrs post-exposure lung tissue was harvested for expression profiling.

**Figure 2:**
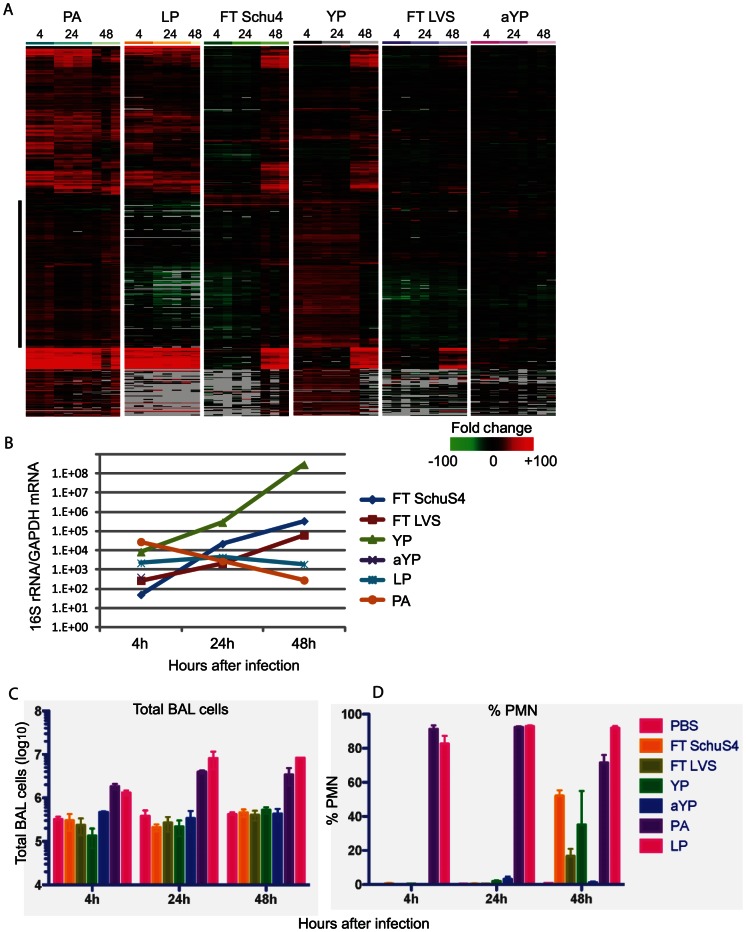
FT SchuS4 induces a limited pulmonary inflammatory response relative to other respiratory pathogens despite active bacterial replication during acute infection. **A.** Expression profiles of inflammatory mediators showing differential expression (at least 2-fold in median expression level, p value<0.01, in at least 1 experimental condition) in lung tissue of animals exposed to respiratory pathogens. Each column represents gene expression data from an individual experiment comparing RNA from lung tissue from an infected animal to pooled RNA from lung tissue from mock-infected animals (n = 9). Genes shown in red were up-regulated, genes shown in green were down-regulated and genes in black indicate no change in expression in infected relative to uninfected animals. Grey indicates no data. **B**. Levels of bacteria present in lung tissue represented by quantitation of 16S rRNA levels. Quantitation of total (**C**) and PMN (**D**) cells present in broncoalveolar lavage fluid from animals exposed to PBS, FT SchuS4, FT LVS, YP, aYP, LP and PA, respectively.

The expression levels of approximately 1400 genes known to be involved in inflammation, including IFN signaling, chemokine/cytokine, antigen presentation, and immune cell activation, were examined in the lungs of animals to specifically characterize the pulmonary inflammatory response to each infection. As shown in Figure 2A, each pathogen was associated with a unique pattern of expression of these transcripts, suggesting differences in activation and/or modulation of host inflammatory networks. Both LP and PA, which cause acute, self-resolving infections, induce an inflammatory response by four hours as evidenced by increased expression of numerous inflammatory mediators, although the induction in response to PA is much more extensive than that to LP (Figure 2A). In general, induction of inflammatory-related genes remained consistent through-out PA infection. There was a slight decline at 48 hrs coinciding with decreased levels of bacterial counts, as measured by qPCR detection of 16S rRNA, in the lungs of animals, possibly a reflection of immune-mediated clearance (Figure 2B). LP-induced expression of inflammatory mediators was also maintained throughout the time-course, as were levels of bacteria present in lungs (Figure 2B). Interestingly, a subset of sequences strongly induced by PA was not induced at any time-point during LP infection (indicated by black bar,Table S1). Many of these sequences encode chemokines (CCL28), cytokines (including IFNα subtypes 5, 12, 13, IL11, IL13, IL24, IL27, IL30), and receptors (CCR3, IL6ra, 17r, IL21r, IL22ra1) suggesting differential recruitment of immune cell subsets during PA and LP infection. Others are key regulators of inflammation and stress response (including TOLLIP, Cd3e, Adora2a, Ltb, Ltbp3, Mef2d). This suggests that while both LP and PA cause acute, self-resolving infection, the cellular mechanisms by which the immune response mediates clearance of bacteria differ between the two pathogens.

In contrast, animals exposed to FT SchuS4 showed a lack of induction of the majority of immune-related genes during the acute phase (<24 hrs) compared to what was observed during LP and PA infection (Figure 2A), with only a limited group of genes showing increased expression. During this time, levels of FT SchuS4 in the lung increased significantly, approximately 3 logs, and by 24 hrs were higher than either LP or PA at the same time-point (Figure 2B). However, by 48 hrs post-exposure there was increased expression of the majority of genes examined to a level and pattern comparable to what was observed during PA infection. This induction correlated with a much smaller increase in bacteria levels between 24 and 48 hrs, suggesting that once activated, inflammatory pathways are capable of slowing FT SchuS4 replication to a certain extent.

Interestingly, despite employing multiple mechanisms to antagonize innate immune responses [24]–[26], YP infection was associated with increased expression of many immune-related genes throughout infection, although the pattern of expression changed between 24 and 48 hrs. At early time-points (4 and 24 hrs) there was a strong induction of one group of genes which was subsequently suppressed by 48 hrs (Figure 2A). Many of these genes were common to those showing increased expression during PA but not LP infection. Increased expression of other immune-related sequences during YP infection was delayed until 48 hrs. Interestingly, this temporal regulation of different groups of immune-related genes correlated with differences in bacterial growth kinetics. The levels of YP in the lungs of exposed animals increased approximately 10-fold between 4 and 24 hrs, but 1000-fold between 24 and 48 hrs, suggesting that the genes induced at 4 and 24 hrs are more effective at controlling YP replication than those induced at later time-points. The decreased expression of these genes at 48 hrs may be a reflection of specific targeting by *Yersinia*.

Similar to what was observed during FT SchuS4 infection, limited induction of inflammatory mediators was observed in the lungs of animals exposed to either attenuated FT LVS or aYP at either 4 or 24 hrs (Figure 2A). The expression levels of a limited number of genes increased by 48 hrs in FT LVS-infected animals. However, the levels are generally less than what was observed during FT SchuS4 infection at the same time-point. Furthermore, while FT SchuS4 infection induced an extensive host transcriptional response at all time-points examined, FT LVS infection was associated with very little change in host gene expression for at least the initial 48 hrs (data not shown). Animals exposed to aYP demonstrated an induction of a slightly larger group of inflammatory mediators than seen in FT LVS-exposed animals, although to similarly low levels (Figure 2A). This is possibly related to poor replication in the lungs of exposed animals as aYP was only detectable at the earliest time-point (Figure 2B).

**Figure 3:**
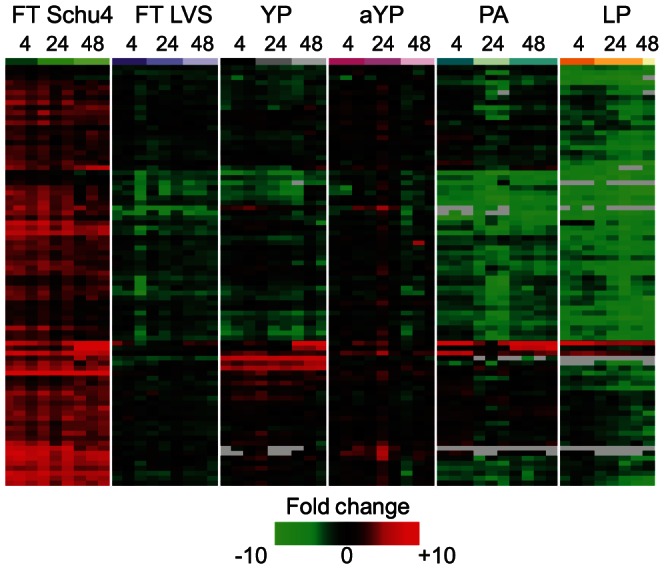
FT SchuS4 induces the expression of a unique subset of inflammatory mediators. Expression profiles of transcripts encoding inflammatory mediators that were regulated (at least 2-fold difference in median expression levels, p-value<0.01) at four hours post-FT SchuS4 exposure. Each column represents expression data from an individual experiment comparing RNA from lung tissue from an infected animal to pooled RNA from lung tissue from mock-infected animals (n = 9). Genes shown in red were up-regulated, genes shown in green were down-regulated and genes in black indicate no change in expression in infected relative to uninfected animals. Grey indicates no data.

The cellular infiltrate present in bronchoalveolar lavage fluid (BALF) was examined to further characterize the pulmonary inflammatory response following aerosol exposure of these pathogens. Animals exposed to either PA or LP demonstrated increased levels of total immune cells present in BALF relative to control animals by 4 hrs post-exposure which was further increased at 24 and 48 hrs (Figure 2C). The composition of this infiltrate was primarily comprised of polymorphonuclear cells (PMNs) (Figure 2D). FT SchuS4-exposed animals show no significant elevation of total immune cells at any time-point relative to control animals, although by 48 hrs PMNs comprised an increased percentage of the cellular composition (Figure 3C and D). This is in agreement with a previous study demonstrating infiltration of PMNs into the lungs of FT SchuS4-exposed animals at later time-points [27]. Similarly YP-infected animals showed an increased percentage of PMNs at 48 hrs post-exposure (Figure 2D).

In summary, despite active bacterial replication and an extensive host transcriptional response to infection, FT SchuS4 infection was associated with a very limited induction of immune-related genes or infiltration of immune cells during the initial 24 hrs. This was particularly evident when compared to the extensive inflammatory response induced by both PA and LP. This almost complete absence of a pulmonary inflammatory response was unique to *Francisella*, even when compared to *Y. pestis*, another highly lethal pathogen. Previous transcriptional profiling of lung tissue from *Francisella*-exposed mice has also shown little induction of inflammatory genes, however, those studies either examined a small set of genes [22] or, in general, showed little transcriptional response [21], possibly related to much smaller inoculums dose used in those studies. The ability of this pathogen to antagonize activation of inflammation is clearly more extensive than previously reported and possibly includes effects on multiple cell types, including lung epithelial cells.

### Immune-related sequences uniquely induced during FT SchuS4 infection suggest targeting of lymphocyte function and host anti-inflammatory pathways

Interestingly, while animals exposed to FT SchuS4 did not show induction of classical pro-inflammatory mediators such as IL-1, TNF-α, or IL-6, gene ontology analysis of transcripts induced following FT SchuS4 exposure did identify 84 genes involved in immune responses (Figure 3 and Table S2). In contrast, animals exposed to FT LVS did not show induction of the immune-related transcripts induced by FT SchuS4 (Figure 3), raising the possibility that they play a role in the extreme virulence uniquely associated with FT SchuS4. Animals exposed to either YP or aYP showed a more extensive induction, but the number of transcripts as well as the level of induction was still less than what was observed in FT SchuS4-infected animals. Animals exposed to either PA or LP showed an induction of only a small subset of the FT SchuS4-induced immune-related genes, despite both pathogens inducing an extensive induction of many other inflammatory-related genes. Collectively, these results suggest that FT SchuS4 infection is associated with a unique host immune response when compared to a less virulent strain of *F. tularensis*, FT LVS, as well as other respiratory pathogens, including both self-limiting and lethal infections. The lack of induction of these transcripts in response to these pathogens was consistent across all time-points, suggesting it is not simply due to differences in kinetics, but rather fundamental differences in activation of host signaling processes. Furthermore, the majority of these sequences were either not induced in response to virulent *Y. pestis* infection, or induced to a lesser degree, suggesting that while both pathogens are associated with high lethality in mice, the underlying mechanisms of pathogenesis differ. Interestingly, these transcripts are strongly suppressed in the lungs of animals infected with the 1918 pandemic strain of influenza virus (Walters, unpublished data), which has been shown to mediate significant induction of pro-inflammatory mediators and PMN infiltration [28]–[30].

Many of the induced transcripts are expressed in multiple immune cell types and contain binding sites for transcription factors known to be activated by innate signaling pathways, including NFAT (Arcn1, Arl5b, Ets1, Mll3, Npepps, Ppp4r2, Prpf38b, Smurf2, Stag1, Zch2), STAT1 (Csnk1a1,Ifih1), STAT4 (Bzw1, Ifh1), STAT3 (Zhx2), STAT5A (Bzw1, Cdc37l1,Ets1, Usp48), and ATF3 (Usp48, Eif2c2). Consistent with this, by 4 hrs post-exposure, there was also increased expression of the transcription factors NFATc1/NFATc3 as well as genes associated with NFAT signaling (ILF3, Csnk1a1) at four hours post-exposure.

Interestingly, several of the induced genes encode proteins that function to modulate inflammation. These include ADAM17, which in addition to pro-inflammatory effects, also suppresses epithelial cell cytokine synthesis [31] and inhibits early neutrophil infiltration by shedding of L-selectin [32]. Also induced was ROCK1, which functions as a suppressor of inflammatory cell migration by regulating the phosphorylation and stability of PTEN [33] and PTPRC (CD45), a protein tyrosine phosphatase that regulates cytokine receptor signaling by suppressing JAK kinase activity [34], [35]. These genes all function to modulate the inflammatory response to mitigate immune-pathological effects. It is possible that the premature induction of these genes immediately following exposure to FT SchuS4 dampens the pulmonary inflammatory response, at least temporarily, to facilitate replication and dissemination of the bacteria. Furthermore, the activation of host pathways which modulate inflammation could explain the broad level of suppression and/or lack of activation that was observed during FT SchuS4 acute infection. While not impossible, it seems unlikely that the levels of a bacteria-specific factor would be sufficient to facilitate the extensive of inflammation observed even by 4 hrs post-exposure.

In summary, while acute FT SchuS4 infection was not associated with induction of the well- characterized, classical inflammatory mediators, the unique induction of a subset of immune-related transcripts suggest a role for targeting of leukocyte function and anti-inflammatory pathways in the extreme virulence of this pathogen.

### Activation of inflammatory response in FT SchuS4-infected animals correlates with altered bacterial gene expression

Examination of the expression levels of immune-related transcripts suggest that activation of a classical innate immune response occurs between 24 and 48 hrs post-FT SchuS4 exposure (Figure 2A) similar to what was observed in a previous study [21]. This temporal regulation of inflammatory signaling pathways could be caused by FT SchuS4 altering its own transcriptome in response to the lung microenvironment. *F. tularensis* is able to adapt to a wide variety of environments through temporal regulation of gene expression [36]. To determine if significant changes in the bacterial transcriptome correlated with the shift in host transcriptional response, *Francisella*-specific mRNA transcripts present in lung tissue at each time-point were profiled using next-generation sequencing. No bacterial-specific mRNA transcripts were detected at the 4 hr time-point, likely due to the low levels of bacteria present at this time. However, 216 and 1151 transcripts were detected (RPKM normalized counts >10) at 24 and 48 hrs, respectively. The greater number of detectable *Francisella*-specific mRNA transcripts detected at 48 hrs is likely at least partially related to the higher levels of bacterial counts in the lungs (Figure 2B). Of the 216 transcripts detected at 24 hrs, 168 showed decreased levels at 48 hrs, while the remaining showed either increased or constant expression levels. This indicates that *Francisella* continues to adapt to the microenvironment throughout *in vivo* infection by both increasing and decreasing the expression levels of specific genes.

The absence of an innate immune response during the first 24 hrs post-infection is followed by a strong inflammatory response at 48 hrs post-infection, as measured by induction of immune-related gene expression and infiltration of PMNs. Similar to what has been reported, this absence is at least partially attributed to active suppression of TLR4 signaling, as evidenced by failure of FT SchuS4-infected mice to respond to exogenous LPS (data not shown) [9]. It is possible that decreased expression of the bacterial factor(s) suppressing the innate immune response coincides with activation of inflammation in the lungs of infected animals. Therefore, analysis of bacterial gene expression data was focused on transcripts showing a trend of decreased expression between 24 and 48 hrs post-infection. Interestingly, a large number of the bacterial-specific genes detectable at 24 hrs demonstrated this trend, with 168 of the 216 sequences showing decreased abundance at 48 hrs (Table S3). Quantitative PCR analysis of a number of bacterial transcripts demonstrated a good correlation with the sequencing data with respect to relative abundances between 24 and 48 hrs (Figure S1). As bacterial loads in the lungs are increasing during this time, decreased abundance likely reflects transcriptional regulation of these genes.

**Figure 4:**
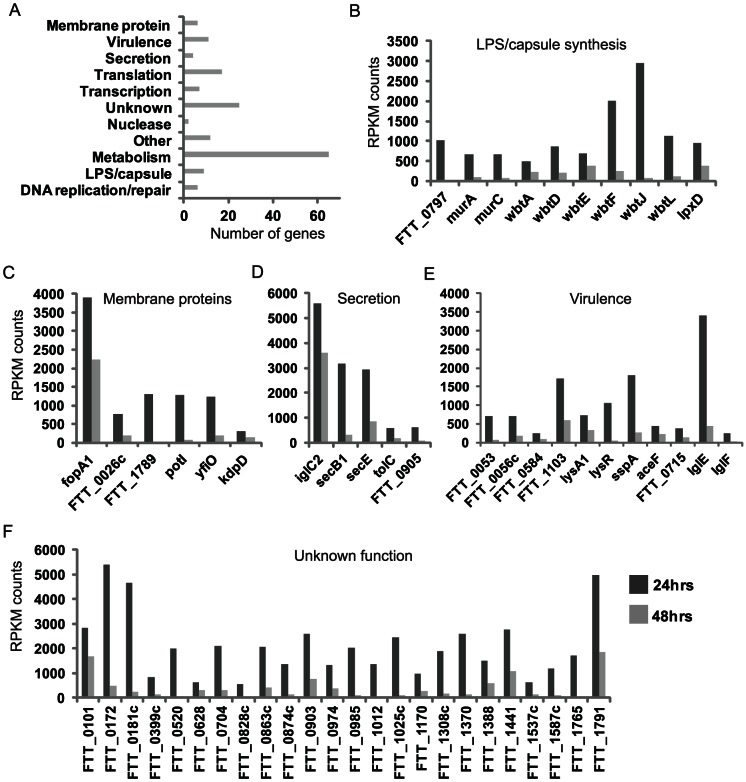
Activation of pulmonary inflammation between 24 and 48 hrs post-FT SchuS4 exposure correlates with altered expression levels of bacteria-specific mRNA transcripts. **A.** Functional distribution of *Francisella* transcripts detected in lung tissue that show decreased abundance at 48 hrs relative to 24 hrs post-exposure. Expression profiles of transcripts that encode genes involved in LPS/capsule synthesis (**B**), membrane proteins (**C**), secretion (**D**), virulence (**E**) and unknown function (**F**) at 24 and 48 hrs post-exposure. Expression levels are shown as normalized (RPMK) counts of detectable reads mapping to FT SchuS4 genome using RNAseq analysis of lung tissue from infected animals. For each run, samples from 3 individual animals were pooled prior to sequencing. * RPMK: reads per kilobase of gene per million reads.

The functional distribution of these sequences is shown in Figure 4A. Of interest are bacterial genes which encode membrane or cell surface components due to their potential ability to interact with cellular signaling components present on the host cell surface or cytoplasm. Many genes showing differential expression levels between 24 and 48 hrs encode surface molecules or proteins involved in the synthesis of such molecules. This includes six genes involved in LPS O-antigen synthesis (wbtADEFJL) (Figure 4B, several of which. (wbtF, wbtJ and wbtL) show a significant decrease in expression level (>1000-fold). Also decreased was the lipid A modifying N-acetyl transferase, IpxD, which was recently found to be responsible for temperature-mediated LPS remodeling [37]. Two other genes, murA and murC, mediate peptidoglycan biosynthesis, an important component of the bacterial cell wall [38]. The hypothetical protein FTT_0797 shares homology to a glycosyl transferase and is part of a gene cluster thought to encode a polysaccharide additional to the lipopolysaccharide O antigen [38].

Decreased expression of membrane proteins was also observed, including fopA1, yfiO, and the putative outer membrane protein (FTT_1789), which has been shown to be specific to *F. tularensis* subsp. *tularensis* using comparative genomic studies (Figure 4C) [39]. FTT_0026c shares homology with major facilitator superfamily of transporters while potl (FTT_0572) is a proton-dependent oligopeptide transport family protein. Perhaps most interesting is KdpD, a sensor histidine kinase that has been shown to phosphorylate the response regulator pmrA. The KdpD/PmrA TCS regulates *Francisella* pathogenicity island (FPI) gene transcription and hence plays an important role in virulence, although the external signals required for activation remain to be identified [36], [40] The differential expression of genes encoding both membrane proteins and those associated with O antigen synthesis may reflect the bacteria remodeling its surface to a more immune-stimulatory phenotype, although the purpose of this remains unclear.

While the genome of *Francisella* is not predicted to encode toxins, it does contain components with homology to Type 1, VI, type IV pilus and Sec-dependent secretion systems which export FPI proteins [41]–[46]. The expression levels of five proteins (IglC, secB1, secE, tolC, FTT_0905) involved in secretion decreased between 24 and 48 hrs, (Figure 4D), suggesting the bacteria may be altering the export of certain factors. FTT_0905 encodes a Type IV pili glycosylation protein transcriptionally regulated by MglA, which controls the expression of the *Francisella* pathogenicity island [47]. Type IV pili-mediated secretion has been shown to modulate PMN infiltration during *Francisella* subsp. *novicida* infection through secretion of the protease PepO [45]. While human pathogenic strains of *Francisella* are predicted to contain an IS-mediated rearrangement which results in a non-secreted form of PepO, it is interesting that reduced expression of a gene encoding another Type IV pili protein (FTT_0905) coincides with infiltration of PMNs in the current study. Furthermore, that FTT_0905 is regulated by MglA suggests a potential role for Type IV pili-mediated secretion in the virulence of pathogenic human *Francisella* strains. TolC is a virulence factor thought to comprise a Type I secretion system, although the secreted factor remains unidentified [44]. The FPI protein IglC, which lacks homology to any characterized bacterial protein, is a potential effector protein exported via a Type VI secretion system [48]. The modulation of multiple genes involved in secretion is particularly interesting as the ability of *Francisella* to antagonize activation of macrophages is thought to be at least partly mediated by a secreted bacterial factor [9].

Also showing a trend for decreased expression were numerous genes previously identified as virulence factors, including those associated with the *Francisella* pathogenicity island (IglE, IglF) (Figure 4E) [48]. SspA, lysA, and lysR are transcription factors implicated in regulating expression of *Francisella* virulence factors [36]. Activation of SspA has been shown to be regulated by the KdpD/PmrA TCS [36]. Others have been shown to be important for *Francisella* virulence (FTT_0053, FTT_0056c, FTT_1103, FTT_0715) but their precise function is not known [49], [50]. Interestingly, FTT_0584 may function to inhibit macrophage cell death during *Francisella* infection [50].

Finally, of the 168 *Francisella*-specific genes showing decreased expression correlating with activation of inflammation in the lungs of infected animals, 24 encode hypothetical proteins or pseudogenes that have previously not been identified as virulence factors (Figure 4D). Many of these do not show significant sequence homology with any other genes in GenBank and so their function cannot be inferred. Some (FTT_1308c, 1791, 0172 and 0181c) comprise sequences which are specific to the highly virulent Type A strain of *Francisella* [39] and so are of particular interest as suppression of TLR4 signaling is specific to this strain (data not shown). Complete sequencing of highly virulent *F. tularensis* strains has revealed little about the identity of possible virulence factors [38]. The ability to correlate bacterial gene expression patterns with phenotypes during *in vivo* infection, such as modulation of inflammatory pathways, may provide insight into the potential function of unannotated *F. tularensis* genes. It is feasible that one or more of these sequences encode a factor that is responsible for the extensive suppression of innate immune response signaling observed during the initial 24 hrs post-exposure. The use of transposon-generated mutant libraries of *F. tularensis* and/or gene-specific targeted mutagenesis will undoubtedly be of use in examining this possibility.

## Discussion

This study represents the first comparison of the global transcriptional response in lung tissue of mice exposed to highly virulent *F. tularensis* ssp. *tularensis* to other respiratory pathogens including the less virulent though lethal *F. tularensis* ssp. *holarctica* LVS, virulent and avirulent *Y. pestis*, *P. aeruginosa*, and *L. pneumophilla*. This study also represents the first simultaneous measurement of both host and *F. tularensis* transcriptome changes that occur during *in vivo* infection and highlights the potentially extensive regulation of bacterial gene expression that occurs during mammalian infection.

The ability of a pathogen to antagonize the host innate immune response to facilitate growth and dissemination is often correlated with the level of virulence. Because of this, it is speculated that the extreme virulence associated with *Francisella tularensis* ssp. *tularensis* is related to its ability to delay the activation of a classical inflammatory response. Indeed, several studies have reported that *F. tularensis* infection impairs the function of macrophage/dendritic cells [9], [12], [14] and little induction of certain sets of immune-related genes has been observed in transcriptional profiling of lung tissue from infected animals [21]–[23]. Significantly, the results of the current study indicate that this antagonism of the innate immune response is much broader and occurs earlier than previously reported, as shown by the lack of induction of an extensive list of known immune-related transcripts. This was particularly apparent when the expression profiles of immune-related genes in FT SchuS4-infected animals were compared to those induced following exposure to either *P. aeruginosa* or *L. pneumophila*. Both of these pathogens cause acute, non-lethal infection that is associated with extensive induction of inflammation-related genes in the lung tissue of exposed animals. Even exposure to *Y. pestis*, a highly lethal pathogen that employs multiple mechanisms to antagonize activation of innate immune signaling, was associated with extensive induction of immune-related genes. The fact that this lack of activation of inflammation is observed in whole lung tissue suggests that *Francisella* is able to suppress innate signaling pathways which function in multiple cell types, possibly including lung epithelial cells and endothelial cells. To date much of the research has focused on *Francisella's* ability to antagonize function/activation of macrophages. While macrophages are central to the innate response to infection, resident epithelial cells have also been shown to play an important role through initiating immune cell infiltration via chemokine expression [51]. Furthermore, a recent study using a mutant defective for replication in macrophages retained its virulence in a murine model of pneumonic tularemia [52]. This suggests that the virulence of *Francisella* is not restricted to suppression of macrophage function and also illustrates the need for more studies focused on the bacteria's effect on other target cells, including lung epithelial cells

The mechanism by which *F. tularensis* mediates this profound suppression of pro-inflammatory pathways is of significant interest, both with respect to understanding the extreme virulence of this pathogen as well as potentially gaining insight into regulatory networks responsible for dampening inflammation. Many mechanisms have been proposed including bacterial expression of catalase, katG,, which serves to harness reactive oxygen species (ROS) generated in cells infected with virulent *F. tularensis* [13], [53]. Multiple studies have implicated the PI3K/Akt pathway in *F.tularensis*-mediated suppression of cytokine production, although results are conflicting about whether the pathway is activated or suppressed [13], [54]–[56]. There is also evidence that the absence of CD14 on alveolar macrophages contributes to the evasion of innate immunity by virulent *F. tularensis*, although the significance of this is unclear as animals lacking CD14 do not exhibit altered susceptibility to pulmonary infection [57].

The lack of increased expression of many key inflammatory mediators during acute *F. tularensis* infection could also be mediated through activation of host pathways that function to dampen/resolve the inflammatory response. Such pathways play a critical role in preventing immunopathology due to uncontrolled immune responses. There is precedence for pathogens manipulating these pathways to limit activation of inflammatory pathways and facilitate their own replication, including increased activation of resolution pathways [58], [59], [60],[61]. Certainly, the global suppression of chemokines and lack of PMN infiltration observed in the lungs of animals exposed to *F. tularensis* in the current study is consistent with activation of these resolution pathways. *F. tularensis* has been shown to inhibit the pro-inflammatory response of endothelial cells via direct contact with the endothelial protein C receptor (EPCR), thus mimicking the anti-inflammatory signaling cascades mediated by activated protein C [62]. The suppression of inflammation during acute *F. tularensis* infection has also been linked to induction of anti-inflammatory cytokines including IL-10 and TGF-β [9], although this was not found in subsequent studies [12]. In the current study, increased levels of IL-10, TGF-*B* and SOCS2/3 were not observed until 48 hrs post-exposure, at which time a significant inflammatory response was observed. However, the unique induction of a subset of immune-related genes in animals exposed to virulent *F. tularensis* points to a potential role in regulating inflammation. The expression levels of these genes appear to be inversely related to overall strength of inflammatory response during infection, at least with respect to the limited repertoire of pathogens used in the current study, suggesting a potential role in dampening the innate immune response. Indeed, at least several of the transcripts have already been shown to modulate the inflammatory response, including ADAM17, ROCK1 and PTPRC. The function of the remaining transcripts warrants further investigation, both to provide more insight into the intricate regulatory networks governing inflammation as well as to identify potential targets for therapeutic intervention in inflammatory disorder.

While many studies have focused on how *Francisella* modulates macrophage/dendritic cell function, less is known about how it may modulate leukocyte populations, including natural killer cells which play a key role in the innate response to intracellular pathogens. Of the limited number of immune-related genes that did show increased expression during acute *Francisella* infection, many are involved in lymphocyte activation/function, including NFAT signaling. This possibly explains the significantly higher level of IFN-γ in FT Schu4-exposed animals compared to those exposed to other respiratory pathogens (data not shown). Involvement of IFN-γ and IFN-γ-inducible genes was also observed in the lungs of mice exposed to both the SchuS4 strain [22] as well as a different type A strain of *Francisella*, FSCO33 [21]. IFN-γ-producing NK cells are known to be present following infection of mice with FT LVS [63]–[65] and in humans following vaccination with inactivated FT LVS [66]. IFN-γ appears to play a key role in protection against FT LVS infection [67]–[69] although NK cells themselves appear to be dispensable. This could be explained by *Francisella* specifically antagonizing the normal function of this immune cell population [65], [70].

FT-SchuS4 infection was characterized by extensive suppression of the majority of measured immune-related genes during the first 24 hrs post-exposure followed by activation of inflammatory pathways to a pattern and level comparable to that of non-lethal respiratory infections. Increased expression of immune-related transcripts and infiltration of neutrophils coincides with an apparent slower growth of bacteria in the lung, suggesting that once activated, the inflammatory response is capable of slowing *Francisella* growth. The mechanism responsible for this activation of inflammation is unclear. One possibility is *Francisella* alters its surface to be more detectable by host innate immune pathways. Characterization of *F. tularenesis*-specific mRNA transcripts by deep sequencing revealed altered expression levels of genes encoding bacterial surface componants, including those associated with LPS and O-antigen synthesis/modification One such gene, the lipid A modifying N-acetyl transferase, IpxD, was recently shown to be responsible for temperature-mediated LPS remodeling and lpxD-null mutants were attenuated in mice [37]. *Francisella novicida* strains lacking enzymes required for lipid A carbohydrate modification were also found to be attenuated in mice and elicited higher levels of pro-inflammatory mediators in infected macrophages [71].This is intriguing in light of recent studies demonstrating that *F. tularensis* alters its surface carbohydrates during host adaptation to impede recognition of bacteria by innate sensing pathways and reduce macrophage activation [72]. This remodeling likely plays a critical role in pathogenesis as null mutants of these genes are often associated with attenuation in mouse models [37], [50], [73]. The LPS of *F. tularensis ssp. novicida* has been found to be a poor stimulator of TLR4/2 signaling, which is thought to be due to unique characteristics of the lipid A structure [74]. As the immune-stimulatory studies of *F. tularensis* LPS have only been performed on bacteria cultured in broth, it would be interesting to examine the structure/inflammatory characteristics of LPS purified from bacteria isolated during *in vivo* infection at different time-points.

In addition to alterations to the bacterial surface, activation of inflammation 48 hrs post-FT SchuS4 exposure could be caused by temporal expression of factor(s) that function to modulate immune response pathways. The inhibitory action of *F. tularensis* on DC/macrophage activation has been shown to be at least partially mediated by a secreted factor [9]. Analysis of the bacterial transcriptome in lung tissue from FT SchuS4-infected mice demonstrated decreased levels of numerous genes involved in secretion between 24 and 48 hrs post-exposure, suggesting the bacteria is altering export of certain effector proteins. These genes are part of Type I (TolC,), Type II (secB1, secE), Type IV pili (FTT_0905) and Type VI (IglC) secretion systems that function to export toxins and other virulence factors [38], [45], [75]. Of particular interest is tolC, which encodes an outer membrane protein involved in drug efflux and type 1 protein secretion that has been identified as a critical virulence factor [76]. It is speculated that tolC is required for the secretion of an unidentified factor, via the type I secretion pathway, that is involved in suppression of pro-inflammatory signaling pathways. Decreased expression of tolC at 48 hrs could be an indicator that *Francisella* is regulating the export of factors which target host defense pathways.

An important finding of this study was that the nearly complete absence of activation of host innate signaling pathways during acute infection was unique to *Francisella*. Furthermore, the active suppression of TLR4 signaling was specific to the FT SchuS4 strain. Because of this, it is logical to assume that *Francisella* encodes unique effectors which mediate these suppressive effects. Many of the bacterial genes showing decreased expression have unknown functions and no homology to other proteins. Some of these, including FTT_1308c, FTT_1791, FTT_0172 and FTT_0181c, are unique to the highly virulent Type A *Francisella* strains. FTT_0172, FTT_0181c and FTT_1791 are among the highest expressed bacterial transcripts detected (approximately 5000 RPMK counts) at 24 hrs and then decrease significantly at 48 hrs. While they have not previously been identified as virulence factors, the correlation of decreased expression with induction of immune-related genes suggests they could be potential candidates as antagonists to innate immune pathways.

Temporal regulation of inflammatory pathways may serve to limit early activation of host defense responses that would eliminate *Francisella*, ensure survival of target cells to allow sufficient replication and also to facilitate release of intracellular bacteria. Many pathogens trigger activation of the inflammasome, a multiprotein platform that plays a key role in host defense by controlling the release of the pro-inflammatory cytokines IL-1B and IL18, bacterial degradation and pyroptosis [77]. Interestingly, *F. tularensis* subsp. *novicida* strains deficient for two membrane proteins found to have decreased levels between 24 and 48 hrs, FTT_584 and fopA, are hypercytotoxic for macrophages [78]. This was linked to increased activation of AIM-mediated pyroptosis and expression of pro-inflammatory cytokines, suggesting that these bacterial products are targeting activation of the inflammasome. Similarly, the tolC deletion mutant is hypercytotoxic to mouse macrophages, elicits higher levels of pro-inflammatory cytokines, and is associated with decreased bacterial burdens in multiple tissues [76]. That these deletion mutants are associated with enhanced cytotoxicity, and in some cases reduced virulence in mice, suggests that *Francisella*-mediated suppression of specific innate immune responses may serve to delay death of key target cells required for replication/dissemination of the bacteria. Following exponential growth in the cytosol of macrophages, *Francisella* may then decrease expression of proteins which inhibit inflammasome activation to allow induction of pyroptosis and subsequent release of bacteria.

Collectively, the results of the current study highlight potentially important alterations in bacterial gene expression which occur throughout *in vivo* infection. Furthermore, it identifies candidate bacterial factors for future investigation into mechanisms of *Francisella*-mediated antagonism of host inflammatory responses. While the analysis was focused on bacterial genes with decreasing expression levels correlating with activation of inflammation, it is important to note that many others increased in response to *in vivo* environment and may also play a significant role in pathogenesis. This highlights the importance of investigating not only the host response to infection, but also the pathogen's response to the *in vivo* environment and how that is potentially altered throughout infection.

## Supporting Information

Figure S1 **Expression levels of** ***Francisella tularensis*** **genes in mouse lung tissue as measured by qRT-PCR.**
(EPS)

Table S1 **Immune-related genes induced during** ***P. aeruginosa*** **but not** ***L. pneumophila*** **infection.**
(XLSX)

Table S2 **Immune-related genes induced during Type A** ***F. tularensis*** **infection.**
(XLSX)

Table S3 ***Francisella*** **-specific mRNA transcripts showing decreased abundance between 24 and 48 hrs in lung tissue from infected mice.**
(XLSX)
